# Synthesis of Poly(ε-caprolactone)-Based Miktoarm Star Copolymers through ROP, SA ATRC, and ATRP

**DOI:** 10.3390/polym10080858

**Published:** 2018-08-02

**Authors:** Venkatesan Sathesh, Jem-Kun Chen, Chi-Jung Chang, Junko Aimi, Zong-Cheng Chen, Yu-Chih Hsu, Yi-Shen Huang, Chih-Feng Huang

**Affiliations:** 1 Department of Chemical Engineering, National Chung Hsing University, 145 Xingda Road, South District, Taichung 40227, Taiwan; satheshyadav10@gmail.com (V.S.); g101065010@gmail.com (Z.-C.C.); berry00713@gmail.com (Y.-C.H.); yishen617@gmail.com (Y.-S.H.); 2 Department of Materials Science and Engineering, National Taiwan University of Science and Technology, 43 Sec. 4, Keelung Road, Taipei 10607, Taiwan; jkchen@mail.ntust.edu.tw; 3 Department of Chemical Engineering, Feng Chia University, 100 Wenhwa Road, Seatwen District, Taichung 40724, Taiwan; changcj@fcu.edu.tw; 4 Molecular Design & Function Group, Research Center for Functional Materials, National Institute for Materials Science, 1-2-1 Sengen, Tsukuba, Ibaraki 305-0047, Japan; AIMI.Junko@nims.go.jp; 5 Research Center for Sustainable Energy and Nanotechnology, National Chung Hsing University, 145 Xingda Road, South District, Taichung 40227, Taiwan

**Keywords:** miktoarm star copolymers, ring-opening polymerization, styrenics-assisted atom transfer radical coupling, atom transfer radical polymerization

## Abstract

The synthesis of novel branched/star copolymers which possess unique physical properties is highly desirable. Herein, a novel strategy was demonstrated to synthesize poly(ε-caprolactone) (PCL) based miktoarm star (μ-star) copolymers by combining ring-opening polymerization (ROP), styrenics-assisted atom transfer radical coupling (SA ATRC), and atom transfer radical polymerization (ATRP). From the analyses of gel permeation chromatography (GPC), proton nuclear magnetic resonance (^1^H NMR), and matrix-assisted laser desorption/ionization time-of-flight mass spectrometry (MALDI-TOF MS), well-defined PCL-μ-PSt (PSt: polystyrene), and PCL-μ-PtBA (PtBA: poly(*tert*-butyl acrylate) μ-star copolymers were successfully obtained. By using atomic force microscopy (AFM), interestingly, our preliminary examinations of the μ-star copolymers showed a spherical structure with diameters of ca. 250 and 45 nm, respectively. We successfully employed combinations of synthetic techniques including ROP, SA ATRC, and ATRP with high effectiveness to synthesize PCL-based μ-star copolymers.

## 1. Introduction

In the past decades, an unprecedented range of (co)polymers were synthesized via various living polymerizations (LPs) containing ring opening (metathesis) polymerization (RO(M)P) [1,2,3], reversible-deactivation radical polymerization (RDRP) [4,5,6,7,8,9,10], chain-growth condensation polymerization (CGCP) [11,12,13,14,15,16], and ionic polymerization [17,18,19,20]. Among these various methods of LPs, RDRP is one of the most powerful and facile tools, mainly comprising reversible addition-fragmentation chain transfer (RAFT) polymerization [21,22,23,24], nitroxide-mediated radical polymerization (NMRP) [5], and atom transfer radical polymerization (ATRP) [25,26], to create diverse polymeric architectures. Thus, polymer science has seen the emergence of various novel polymer architectures—such as homo/block star (co)polymers, dumbbell/H-shape branched copolymers, dendritic copolymers, and star copolymers—for a variety of applications. Among the nonlinear polymers, the synthesis of star or star-shape (co)polymers are in particular of great interests because of the structural flexibility of arm designs and the diversity of the central core moieties to afford star homo/random/block/gradient copolymers, miktoarm star copolymer, etc. Thus, there is a high demand to achieve a high degree of control over the polymer architectures via facile and inexpensive procedures.

Star polymers are generally synthesized through “grafting onto”, “core-first”, or “arm-first” methods [27,28,29]. In the “grafting onto” strategy, convergent reactions of polymer chains with a reactive chain end group and a multi-functional core are quantitatively proceeded to afford star polymers. The drawback of this method comes from the mobility of the polymer chains, for example chain rigidity and high molecular weight, etc. that lead to slow and inefficient reactions. A facile and efficient organic reaction is in high demand. In the “core-first” strategy, an initiating core with multi-functionality is synthesized first followed by the polymerization of a monomer from the core to obtain star polymers with defined numbers of arms. The resulting chain end of each arm can undergo subsequent chain extension with another monomer to afford star block copolymers. In the “arm-first” strategy, the reactive arms are synthesized first followed by facilely gathering the reactive site of the arms with cross-linkers to form star polymers. However, the resulting star polymers have a statistical distribution in the number of arms. A variety of star polymers were synthesized based on the aforementioned methods. In examples of the “grafting onto” strategy, efficient organic reactions of copper(I)-catalyzed alkyne-azide cycloaddition (CuAAC) reaction [30,31] and Diels–Alder (DA) reaction [32] were used to synthesize different types of star polymers. In examples of the “core-first” strategy, Huang et al. described the synthesis of tetra-functionalized adamantane initiator and proceeded with ATRP to obtain a PMMA star polymer [33]. The compatibility of linear and star PMMA with phenolic blends were investigated [34]. The same group developed hyperbranched core star polymers by using an active-site-transfer methodology in a one-pot manner [35]. Alonzo et al. reported a series of amphiphilic star block copolymers exhibiting cores of PSt and coronas of poly(2-vinyl pyridine) (P2VP) through sequential anionic polymerizations in a systematic kinetics preferential absorption method [36]. In examples of the “arm-first” strategy, Matyjazsewski et al. described the synthesis of PnBA-*b*-PtBA (i.e., *n*- and *tert*-butyl acrylate) star block copolymers via ATRP with a ppm-level copper catalytic system [37]. Aimi and co-workers developed a non-volatile organic memory device based on organic field effect transistor (OFET) technique by using tetra-functionalized metallo-phthalocyanine (MPc) core to obtain polystyrene (PSt) four-arm star polymers via ATRP [38,39]. Grayer and co-workers described the synthesis of PSt first followed by growing the P2VP chain from the core to afford mikto-arm star (μ-star) copolymers of the PSt_n_P2VP_n_ (i.e., *n* = 13 and 40 arms) via anionic polymerization [40]. Kakuchi et al. synthesized A_3_B_3_, AB_2_, and AB_3_ μ-star copolymers and demonstrated their interesting physical properties [41,42,43]. Recently, we have reported novel miktoarm star copolymers containing poly(ε-caprolactone) (PCL) and poly(*N*-octyl benzamides) via subsequent CGCP, styrenics-assisted atom transfer radical coupling (SA ATRC), and ROP [44]. From the literature, manipulation/control of the aforementioned three methods are highly desired in order to pursue the synthesis of novel branched/star copolymers that might possess unique physical properties. 

**Scheme 1:**
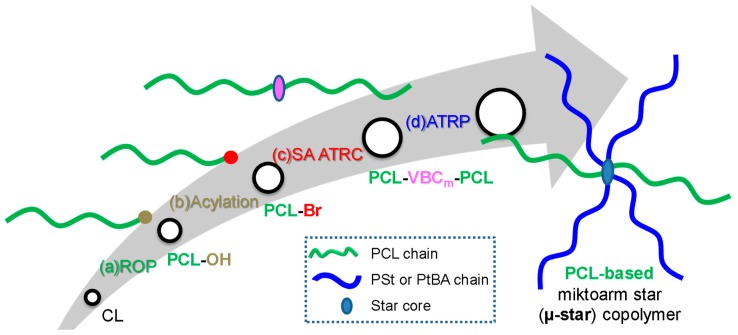
Synthetic routes of poly(ε-caprolactone)-based μ-star copolymers.

In this study, we develop a new strategy for the synthesis of PCL-based μ-star copolymers. As shown in Scheme 1, we carry out the ROP of ε-caprolactone (CL) monomer followed by quantitative chain end modification to afford chain-end functionalization PCL–Br precursor via acylation with 2-bromoisobutyryl bromide. Then SA ATRC of PCL–Br with 4-vinylbenzyl chloride (VBC) was conducted to obtain mid-chain functionalized macroinitiator (PCL-VBC_m_-PCL MI). The PCL-VBC_m_-PCL MI containing ATRP initiating groups of benzylic chloride subsequently was subjected to chain extensions with styrene (St) and tBA monomers to afford PCL-based μ-star copolymers (i.e., PCL-μ-PSt and PCL-μ-PtBA). The physical properties of these copolymers were examined.

## 2. Materials and Methods

### 2.1. Materials

Copper powder (Cu(0), 99.5%, 150 mesh), copper(I) chloride (CuCl, 97%), copper(I) bromide (CuBr, 99%), *N*,*N*,*N*′,*N*″,*N*″-pentamethyldiethylenetriamine (PMDETA), alumina (Al_2_O_3_ neutral), benzyl alcohol (BzOH), 4-vinylbenzyl chloride (VBC), styrene (St), and *tert*-butyl acrylate (tBA, 98%) were purchased from Sigma–Aldrich (St. Louis, MO, USA) . 2-Bromoisobutyryl bromide (BiB, 97%), ε-caprolactone (CL, 98%), tin(II) 2-ethylhexanoate (Sn(oct)_2_), and triethylamine (TEA, 99.5%) were purchased from Acros (Geel, Belgium). All solvents and monomers were purified and stored with dried molecular sieves prior to use.

### 2.2. ROP with ε-Caprolactone and Chain End Modification (Shown in Scheme 1a,b, respectively)

An oven dried 100 mL Schlenk tube and magnetic stirrer bar with BzOH (0.83 mL, 8 mmol), CL (8.86 mL, 80 mmol), and Sn(oct)_2_ (32.40 mg, 0.08 mmol) were charged. The tube was sealed and deoxygenated using freeze–pump–thaw for three cycles and back refilled with N_2_. The set up was placed into preheated oil both at 120 °C for 24 h. The reaction mixture was cooled to room temperature in an ice bath, diluted with tetrahydrofuran (THF) and twice re-precipitated into cold methanol (MeOH). The white powders were collected and dried under vacuum to afford poly(ε-caprolactone) with a hydroxyl chain end (PCL–OH; *M*_n_ = 2500 g/mol and PDI (i.e., *M*_w_/*M*_n_) = 1.18 (*M*_n_ ≡ number-average molecular weight and *M*_w_ ≡ weight-average molecular weight); yield = 65%).

A clean and dried 100 mL round-bottom flask was charged with PCL–OH (4.0 g, 1.25 mmol), TEA (1.76 mL, 12.5 mmol), and 40 mL of anhydrous dichloromethane (DCM) and cooled to 0 °C under N_2_ atmosphere. BiB (1.54 mL, 12.5 mmol) was diluted with anhydrous DCM and slowly added to the mixture by a cannula. After complete addition, the solution was stirred at ambient temperature for another 72 h. The undissolved solid was removed by filtration and the resulting solution was quenched with saturated NaHCO_3(aq)_, subsequently washed with brine solution and then dried over MgSO_4_. The solvent was removed under reduced pressure, the obtained oily liquid was re-dissolved in THF. The solution was precipitated in cold MeOH. The resulting solid was collected by filtration and dried under vacuum to yield poly(ε-caprolactone) bearing a terminal isobutyryl bromide group (PCL–Br; *M*_n_ = 3000 g/mol and PDI = 1.14; yield = 76%).

### 2.3. SA ATRC of PCL–Br with VBC (Shown in Scheme 1c)

The coupling PCL-VBC_m_-PCL product was synthesized according to our previous report [15,16,44]. A typical synthetic procedure: A 25 mL Schlenk flask was charged with PCL–Br (0.15 g, 0.05 mmol), VBC (110 μL, 0.76 mmol), PMDETA (30 μL, 0.13 mmol) and dry toluene (2.5 mL). The mixture was deoxygenated using freeze–pump–thaw for three cycles. The mixture was frozen and back-filled with N_2_. CuBr (7.3 mg, 0.05 mmol) and Cu powder (19.3 mg, 0.3 mmol) were added into the flask, proceeded with two additional freeze–pump–thaw cycles and then sealed the flask. At ambient temperature an initial sample was taken via a syringe to trace the coupling reaction. After completion of the reaction, the flask was placed in an ice bath and exposed with ambient atmosphere and the crude product was diluted with THF and passed through a neutral Al_2_O_3_ column. The solution was concentrated under vacuum and twice precipitated into cold MeOH. The resulting white powder was collected and dried under vacuum to afford a poly(ε-caprolactone) with mid-chain functionality of benzylic chlorides (PCL-VBC_m_-PCL; *M*_n_ = 5700 g/mol and PDI = 1.18; yield = 79%).

### 2.4. Chain Extension of PCL-VBC_m_-PCL with St or tBA through ATRP (Shown in Scheme 1d)

An example for ATRP with tBA: an oven dried 25 mL Schlenk tube were charged with PCL-VBC_m_-PCL (0.05 g, 0.018 mmol), tBA (0.53 mL, 3.63 mmol), PMDETA (3.8 μL, 0.02 mmol), and anisole (0.53 mL). The tube was sealed using a rubber septa and filled with nitrogen. The mixture was degassed by means of freeze–pump–thaw cycles and then back-filled with nitrogen. CuCl (1.8 mg, 0.02 mmol) was added and the tube was sealed with a glass stopper. The mixture was kept at 70 °C in a thermostated oil bath. An initial sample was taken and the chain extension was periodically traced by GPC. After reaction completed, the mixture was diluted with THF and passed through a neutral Al_2_O_3_ column to remove the catalyst. The resulting solution was concentrated under vacuum and precipitated into MeOH. The obtained light-yellowish sticky solid was collected and dried under vacuum to afford a miktoarm star copolymer (PCL-μ-PtBA: *M*_n_ = 37,000 g/mol, PDI = 1.50, yield = 70%).

### 2.5. Deprotection of PCL-μ-PtBA

A 10 mL-flask equipped was dried, purged with nitrogen, and charged with PCL-μ-PtBA (100 mg) and dry DCM (5 mL). TFA (2 mL) was added and the mixture was stirred at room temperature for 24 h to remove the *tert*-butyl group. The concentrated crude product was dissolved in DCM and precipitated into hexane to obtain afford PCL-μ-PAA a yellowish-white viscos liquid (32 mg, yield 60%).

### 2.6. Characterization

Gel permeation chromatography (GPC) was performed at 40 °C in tetrahydrofuran (THF) with a flow rate of 1 mL/min, containing a Waters 515 pump, a Waters 486 absorbance detector, a Waters 410 differential refractometer, and two PSS SDV columns (Linear S and 100 Å pore size). The characterization of *M*_n_, *M*_w_, and PDI (i.e., *M*_w_/*M*_n_) was estimated by monodisperse polystyrene (PSt) standards. Proton nuclear magnetic resonance (^1^H NMR) measurements were conducted by using a Bruker 500 NMR in CDCl_3_ and the chemical shift was rectified by setting the internal standard of CDCl_3_ as 7.26 ppm. FT-IR spectra were analyzed by a Nicolet Avatar 320 FT-IR Spectrometer with 16 scans at a resolution of 2 cm^−1^. The dissolved samples were drop-casted onto a KBr disk and dried under vacuum before FT-IR analysis. Matrix-assisted laser desorption/ionization time-of-flight mass spectrometry (MALDI-TOF MS) were recorded on a Bruker Autoflex III spectrometer plus in the reflectron ion mode. 2-(4-Hydroxyphenylazo)benzoic acid (HABA) was used as the matrix with sodium chloride (NaCl) as the cationizing salt for the MALDI-TOF mass measurements. The glass transition temperature (*T*_g_) of the polymers was measured by a Seiko 6220 differential scanning calorimetry (DSC) with a protocol as follows: raising temperature from 25 to 150 °C, keeping 3 min isothermal at 150 °C, and quenching the samples by liquid nitrogen were pre-treated. Then the thermal traces were recorded by heating the sample from 0 to 150 °C (ramp 20 °C/min) under nitrogen. Thermogravimetric analysis (TGA) of the samples was measured by a TA Instruments Q50 analyzer (scan rate: 20 °C/min; from 100 to 800 °C under nitrogen) with a platinum holder. Atomic force microscopy (AFM) was conducted using a Seiko SPA400 microscope. Clearly well-dissolved solutions of pure and μ-star (co)polymer (1 wt % in CH_2_Cl_2_) were drop-casted onto silicon wafers; after the solvent evaporated, the samples were annealed overnight above their *T*_g_ under vacuum. The height and phase images were recorded and accompanied with the height difference (∆H (nm)) and tapping angle phase difference (∆deg (°)) individually. 

## 3. Results and Discussion

**Figure 1:**
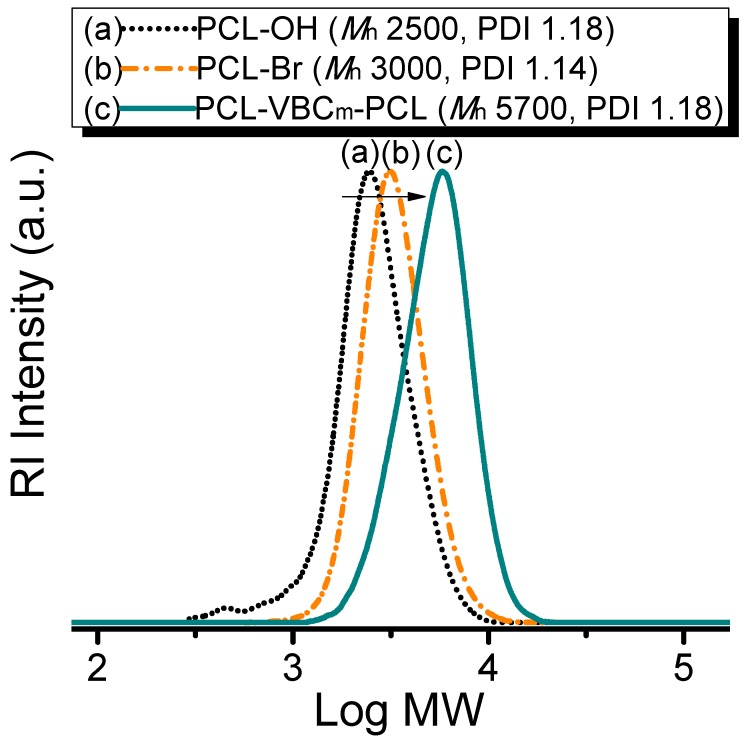
GPC traces (RI detector) of (**a**) PCL–OH, (**b**) PCL–Br, and (**c**) PCL-VBC_m_-PCL.

**Figure 2:**
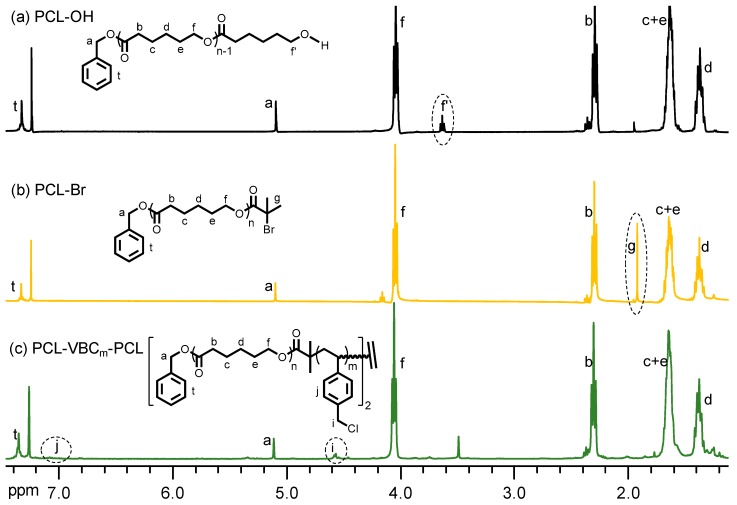
^1^H NMR spectra (400 MHz, CDCl_3_) of (**a**) PCL–OH, (**b**) PCL–Br, and (**c**) PCL-VBC_m_-PCL.

Several examples of using the efficient convergent method of styrenics-assisted atom transfer radical coupling (SA ATRC) were demonstrated for the synthesis of block, macrocyclic, and miktoarm star (μ-star) copolymers [15,16,44,45,46]. Herein, we combine the SA ATRC with ring-opening polymerization (ROP) and atom transfer radical polymerization (ATRP) to synthesize unique PCL-based μ-star copolymers. Figure 1 shows the gel permeation chromatography (GPC) results of (a) PCL–OH, (b) PCL–Br, and (c) PCL-VBC_m_-PCL corresponding to after ROP with CL, chain end modification, and after SA ATRC, respectively. Comparison of the curves for 1a and 1b reveals that the molecular weight (MW) had shifted slightly from 2500 to 3000 g/mol with low PDI values after acylation of PCL–OH chain end. After SA ATRC of PCL–Br with VBC (curve c: VBC/PCL–Br/CuBr/Cu(0)/PMDETA = 15/1/1/6/2.5 at 25 °C), *M*_n_ increased to approximately 5700 g/mol with still remaining a low PDI value (1.18). According to the literature [15,47], the extent of coupling (*x*_c_ = 2 × (1 − *M*_n,0_/*M*_n_) where *M*_n,0_ is the initial number-average molecular weight and *M*_n_ is the value after coupling) was estimated. The trace c in Figure 1 has a high *x*_c_ value of approximately 0.94 with a monomodal MW distribution, implying the high efficiency of SA ATRC at low temperature. The corresponding polymers were analyzed by ^1^H NMR spectroscopy. Figure 2a shows the sample of PCL–OH. The representative signals of PCL repeating units (i.e., peaks b–f) were assigned and the signals from the chain ends were obviously observed (i.e., peaks t, a, and f’ (~7.38, 5.10, and 3.65 ppm, respectively)). After acylation of PCL–OH, the disappearance of peak f’ and appearance of peak g (~1.88 ppm) were shown in Figure 2b, indicating a quantitative chain end modification to afford PCL–Br. After SA ATRC of PCL–Br with VBC, the disappearance of peak g and appearance of peaks i and j (~4.59 and 7.05 ppm, respectively) were revealed in Figure 2c, implying the occurrence of the convergent reaction that inserted a small amount of VBC. 

**Figure 3:**
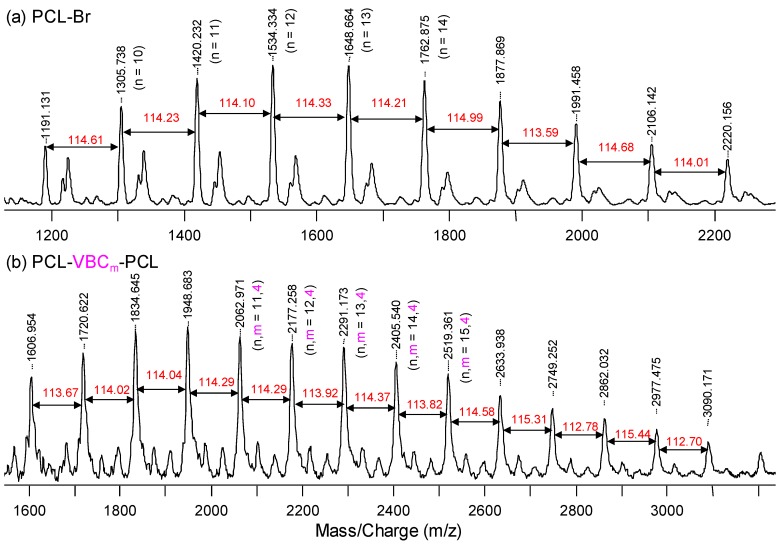
MALDI-TOF MS of (**a**) PCL–Br and (**b**) PCL-VBC_m_-PCL polymers (n: repeating units of CL).

To further understand the chemical structures obtained after SA ATRC, the precursor (PCL–Br) and coupling polymer (PCL-VBC_m_-PCL) were analyzed by MALDI-TOF MS using 2-(4-hydroxyphenylazo)benzoic acid (HABA) as the matrix. Accordingly, we can avoid the influence of the remained precursor on the estimation of the chemical structure. Figure 3a shows the MALDI-TOF spectrum of PCL–Br (*M*_n_ = 3000 g/mol, PDI = 1.14), indicating a series of peaks separated by approximately 114.1 Da, which correspond to the molar mass of the PCL repeating unit. Taking an example of the peak of *m*/*z* = 1420.2 (n = 11), the initial benzylic group of PCL–Br was lost and hydrogenated (i.e., *m*/*z* = head (MW_hydroxyl_ = 17) + n × units (MW_(n__×CL)_ = 11 × 114.1) + end (MW_tertiary bromoester_ = 149.9) = 1421.9). In MALDI-TOF measurements, chain-end cleavages are commonly acquired due to the formation of unstable ions that fragment after acceleration in the ion source region [48,49]. With the addition of NaCl as the cationizing salt, Figure 3b shows the MALDI-TOF spectrum of PCL-VBC_m_-PCL (*M*_n_ = 5700 g/mol, PDI = 1.18) that also displays a series of peaks corresponding to the PCL repeating unit (ca. 114 Da). Taking an example of the peak at *m*/*z* = 2405.5 (n = 14), a coupling product with four VBC inserted units was revealed (i.e., *m*/*z* = chain-ends (MW_hydroxyls_ = 17 × 2) + mid-links (MW_tertiary esters_ = 70 × 2) + *n* × units (MW_(n__×CL)_ = 14 × 114) + m × inserted units (MW_(m×VBC)_ = 4 × 152.6) + Na^+^ (22.9) = 2404.9). The estimated and measured m/z values with different m units of were summarized and compared in Table S1. With the aid of the styrenics as a coupling agent (herein: VBC), high efficiency of SA ATRC for the synthesis of mid-chain functionalized polymers with benzylic chlorides was attained. Our proposed mechanisms of the SA ATRC reaction are illustrated in Scheme S1 and Table S2. The rate constants of activation, deactivation, and termination of PCL–Br were presumably similar to that of nature of ethyl 2-bromoisobutyrate (EBiB) and methyl methacrylate (MMA) due to structural analogy of tertiary bromoester (i.e., *k*_a,EBiB_, *k*_da,EBiB_, and *k*_t,MMA_, respectively). In the presence of VBC, the rate constants were presumably similar to that of benzylic chloride (BzCl), 1-phenyl ethylbromide (PEBr) and styrene (St) (i.e., *k*_a,BzCl_, *k*_da,BzCl_, *k*_a,PEBr_, *k*_da,PEBr_, and *k*_t,St_, respectively). With the absence of VBC, the generated methacrylic macroradical (PCL•) preferred the termination of disproportionation as illustrated in schemes (ES1) and (ES2) and species **I** and **II** were obtained as major products. In the presence of VBC, the PCL macroradical was led to fast addition of VBC. Then the termination mechanism switched from scheme (ES2) to schemes (ES3–ES7). To achieve high coupling efficiency, four successive factors are satisfied [50,51]: (1) moderate equilibrium constant value in scheme (ES1) (*K*_EBiB_ ~ 10^−7^) provides efficient activation/deactivation reactions at low temperature and prolongs the radical life-time; (2) rapid formation of species **III** results from a fast addition rate of methacrylic radical to VBC in scheme (ES3) (*k*_i_ ~ 5 × 10^3^ M^−1^ s^−1^); (3) negligible occurrence of scheme (ES4) arises from the activation rate from the benzylic chloride moiety is much lower than that of the phenyl alkylbromide moiety in the dormant species of PCL-VBC–Br (i.e., *k*_a,PEBr_ (~8.8 × 10^−2^)/*k*_a,BzCl_ (~2.9 × 10^−3^) = 30). (4) At low temperature, the concentration of VBC ([VBC]_0_ = 0.3 M) and propagation rate constant (*k*_p,VBC_ ~ 170 M^−1^ s^−1^) should be sufficiently low to avoid eminent chain extension (i.e., the *x* value of species **IV** in scheme (ES5) is small). With the nature of the styrene radical termination in scheme ES6, fast recombination was mainly conducted (*k*_t,St_ ~ 10^8^ M^−1^ s^−1^) and formed species **V** (i.e., PCL-VBC_4_-PCL product).

**Figure 4:**
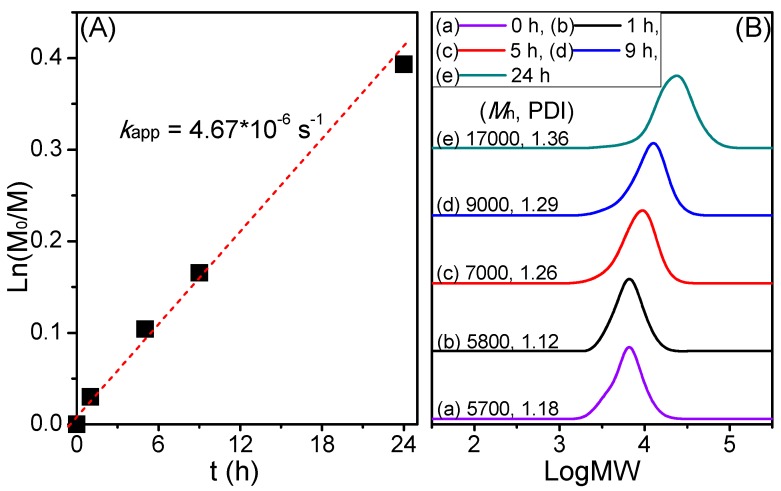
Chain extension of PCL-VBC_4_-PCL with St of (**A**) kinetic plots and (**B**) GPC traces (eluent: THF).

**Figure 5:**
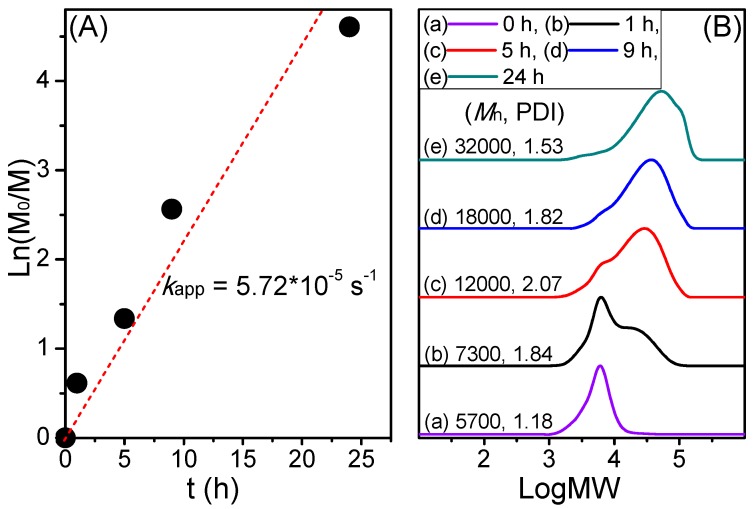
Chain extension of PCL-VBC_4_-PCL with tBA of (**A**) kinetic plots and (**B**) GPC traces (eluent: THF).

The obtained PCL-VBC_4_-PCL polymer was then used as a macroinitiator (MI) for chain extensions through ATRP from the mid-chain functional moiety of benzylic chlorides. Generally, a first order kinetic is examined to interpret the living polymerization fashion through a Ln vs. t plot (i.e., Ln(M_0_/M) = *k*_app_t where M_0_ is the initial concentration of monomer, M is the concentration of monomer at time, and *k*_app_ is the apparent reaction rate constant that is the multiplication of propagation rate constant and concentration of the propagating radical (i.e., *k*_app_ = *k*_p_[M•]) [26]. Figure 4A demonstrates the kinetic plot of the chain extension with St monomer (St/PCL-VBC_4_-PCL/CuCl/PMDETA = 500/1/1/1 in anisole at 70 °C; [St]_0_ = 4.3 M). A first-order kinetic plot showed a linear relationship with a *k*_app_ value of approximately 4.67 × 10^–6^ s^−1^. Figure 4B displays the GPC traces of the corresponding periods of reaction time. A gradual growth of MWs (values of *M*_n_ increased from 5.8 k to 17 k g/mol) with monomodal traces and low PDIs (<1.4) were observed during the chain extension. The results indicated a typical controlled/living radical polymerization fashion and we successfully synthesized PCL_2_-μ-PSt_4_ miktoarm star copolymers. Figure 5A displays the kinetic plot of the chain extension with tBA monomer (tBA/PCL-VBC_4_-PCL/CuCl/PMDETA = 200/1/1/1 in anisole at 70 °C; [tBA]_0_ = 3.4 M). A first-order kinetic plot showed a linear relationship with a *k*_app_ value of approximately 5.72 × 10^–5^ s^−1^ that is approximately an order magnitude faster than that of the St case. Due to the fast propagation rate of tBA at 70 °C (*k*_p,tBA_ ~ 4.2 × 10^4^ M^−1^ s^−1^), however, a gradual growth of MWs (*M*_n_ ~ 7.3 k–32 k g/mol) with bimodal traces and high PDIs (>1.81) was obtained. Meanwhile, with chain extension, the lower MW peaks with nearly stationary was ascribed to the presence of MI that possesses low activation rate constant at 70 °C (*k*_a,BzCl_ ~ 1.8 × 10^–2^ M^−1^ s^−1^) [51]. At the later stage of chain extension, eventually, a PCL_2_-μ-PtBA_4_ miktoarm star copolymer was obtained (*M*_n_ ~ 32 k g/mol and PDI = 1.53).

**Figure 6:**
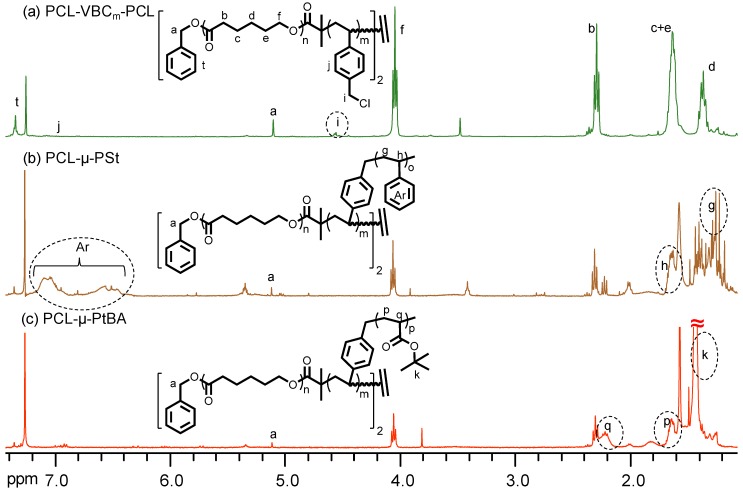
^1^H NMR spectra (400 MHz, CDCl_3_) of (**a**) PCL-VBC_m_-PCL, (**b**) PCL-μ-PSt, and (**c**) PCL-μ-PtBA (co)polymers.

The resulting products of PCL-μ-PSt and PCL-μ-PtBA were subjected to structural elucidation by using ^1^H NMR spectroscopy. Figure 6a shows the spectrum of PCL-VBC_m_-PCL MI that possesses methylene group of peak i (~4.5 ppm). In PCL-μ-PSt (Figure 6b), besides the representative signals from the PCL backbone, the disappearance of peak i and appearance of peaks Ar, h, and g from phenyl ring and PSt backbone (~6.6–7.1, 1.7, 1.2 ppm, respectively) were acquired. In PCL-μ-PtBA (Figure 6c), similarly, the disappearance of peak i and appearance of peaks q, p, and k from PtBA backbone (~2.1, 1.6, 1.4 ppm, respectively) were obtained. FT-IR spectra of the PCL, PCL-μ-PSt, and PCL-μ-PtBA were shown in Figure S1. The results supported the chain extensions of PSt and PtBA chains from the PCL-VBC_m_-PCL MI.

**Figure 7:**
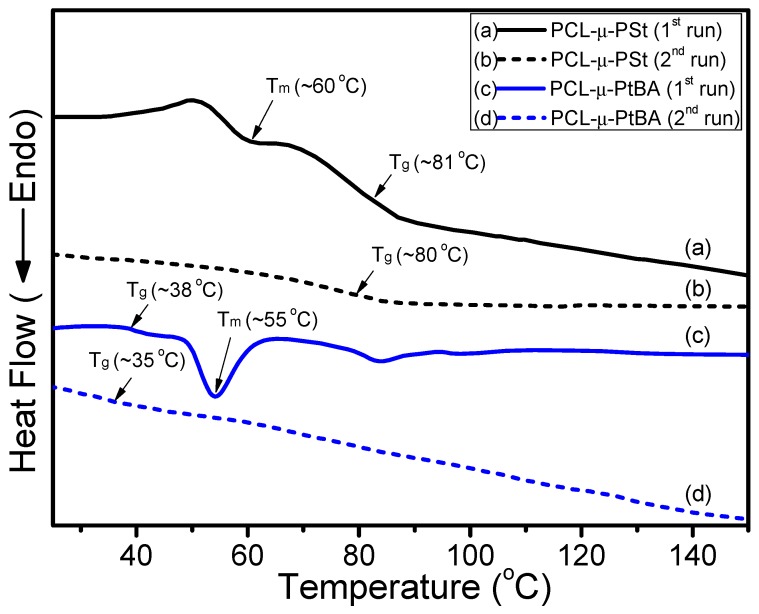
DSC traces of (**a**,**b**) PCL-μ-PSt and (**c**,**d**) PCL-μ-PtBA star copolymers (**a**,**c**: first run and **b**,**d**: second run).

**Figure 8:**
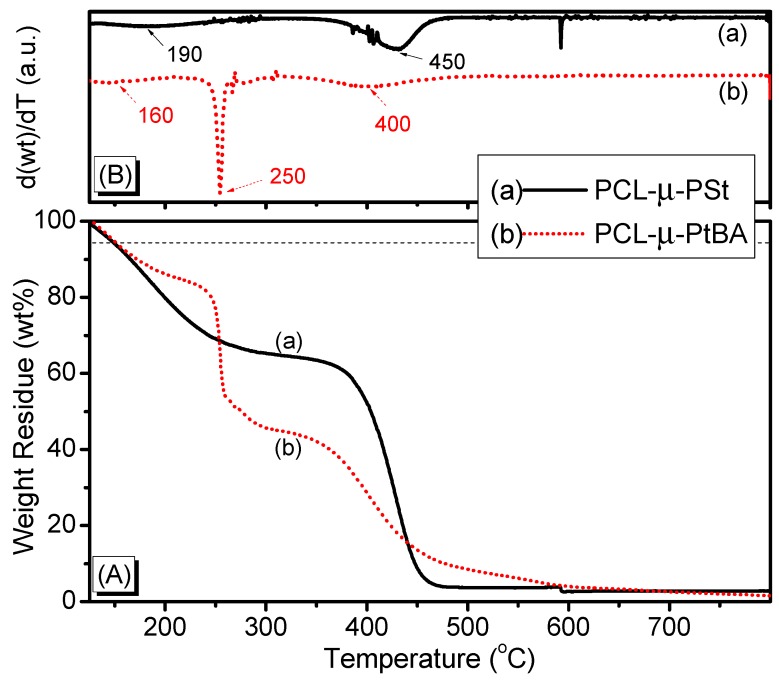
TGA (**A**) and DTGA (**B**) traces of (**a**) PCL-μ-PSt and (**b**) PCL-μ-PtBA star copolymers.

Thermal properties of the μ-star copolymers were measured by differential scanning calorimetry (DSC) and thermogravimetric analysis (TGA) with a heating rate of 20 °C/min under nitrogen. Figure 7 shows the two-run DSC traces of the PCL-μ-PSt and PCL-μ-PtBA copolymers. In the first runs of PCL-μ-PSt and PCL-μ-PtBA (solid lines of a and c), the *T*_g_s of PSt and PtBA chains were respectively observed at approximately 81 and 38 °C. In addition, the *T*_m_ peak of PCL with low intensity can be also detected at approximately 57 °C in each case, indicating the presence of PCL crystalline in the PCL-μ-PSt and PCL-μ-PtBA star copolymers. In the second runs (dash lines of b and d), the *T*_g_s of PSt and PtBA chains were still observed but the *T*_m_ of PCL was disappeared in each case. Before the second run, notably, the samples were heated to 150 °C and quenching to 0 °C. With a fast quenching step, the disappearance of melting behavior indicated a slow crystallization rate of PCL segments, resulting from the nature of the star polymer with restricted chain mobility [52]. Figure 8 displays the TGA analysis of the PCL-μ-PSt and PCL-μ-PtBA copolymers. The temperature of 5 wt % loss of sample (*T*_d5%_) were both approximately 150 °C in curves a and b in Figure 8A. Then, we further differentiated the traces to comprehend the degrading steps in each curve. In curve a of Figure 8B, the first degradation peak was ca. 190 °C and the second degradation peak was ca. 450 °C which were mainly due to the degradation of PCL and PSt segments, respectively. In curve b of Figure 8B, a three-step degradation behavior was observed (ca. 160, 250, and 400 °C), indicating the corresponding degradations of the PCL segment, *tert*-butyl group, and PtBA backbone, respectively. Thermal properties of these miktoarm star copolymers are summarized in Table 1.

**Figure 9:**
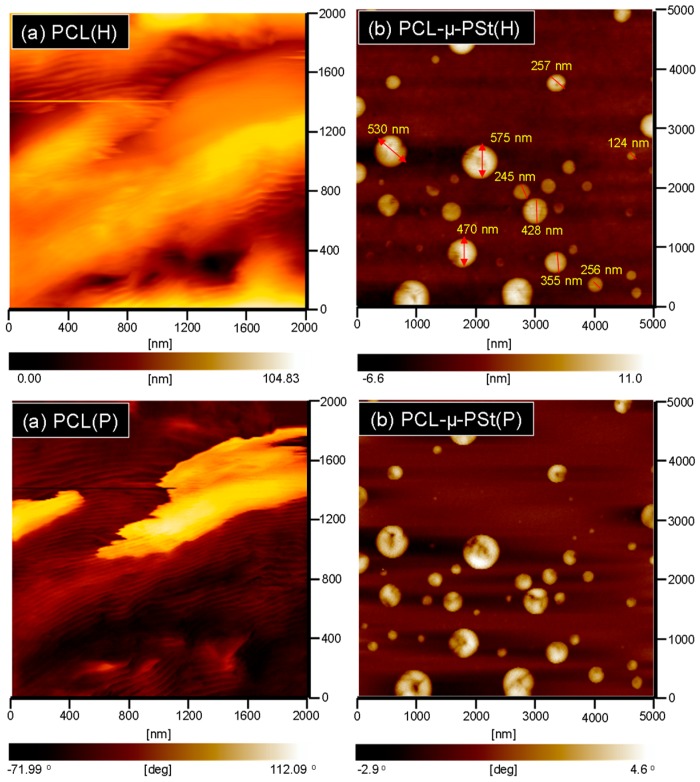
AFM images of (**a**) PCL and (**b**) PCL-μ-PSt (co)polymers (H: height and P: phase).

**Figure 10:**
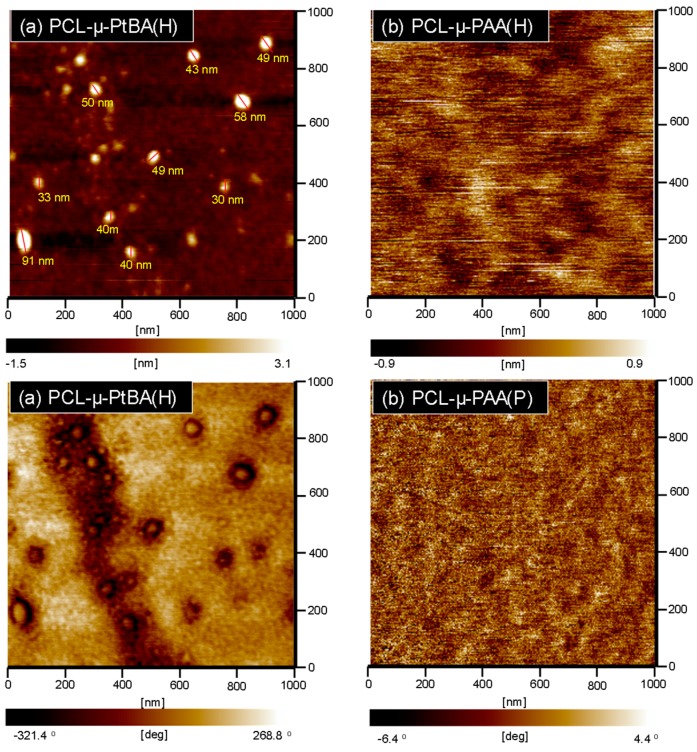
AFM images of (**a**) PCL-μ-PtBA and (**b**) PCL-μ-PAA star copolymers (H: height and P: phase).

**Figure 11:**
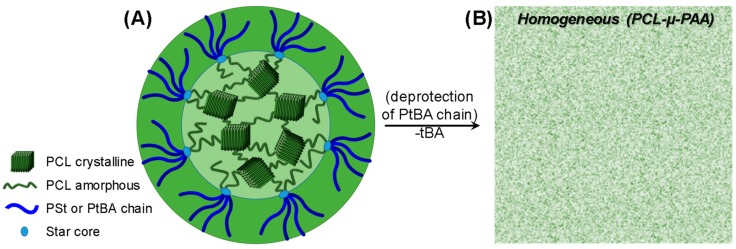
Proposed self-assembled microstructures of μ-star copolymers: schematic representation of (**A**) self-assembled microstructure from immiscible segments and (**B**) homogeneous surface from miscible segments.

Miktoarm star polymers were then dissolved in CH_2_Cl_2_ (2 wt %) and drop-casted onto silicon wafers to examine their topology by using AFM. Figure 9 demonstrated the topological difference of PCL and PCL-μ-PSt (co)polymers with height (H) and phase (P) images. In pure PCL (Figure 9a), the images displayed morphological heterogeneity in their height and phase. The difference was because of the amorphous and crystalline macrophases of PCL. In the PCL-μ-PSt copolymer (Figure 9b), an interesting nanoparticle morphology with an average diameter of approximately 250 nm was obtained. The presence of nanoparticles presumably resulted from the self-assembly of the PCL-μ-PSt copolymer. On the other hand, the *tert*-butyl groups of the PCL-μ-PtBA copolymer were subjected to deprotection with the use of TFA (trifluoroacetic acid) to afford PCL-μ-PAA (PAA: polyacrylic acid) copolymer. FT-IR of the resulting copolymers were shown Figure S2. Figure 10 displayed the AFM results of PCL-μ-PtBA and PCL-μ-PAA copolymers. In the PCL-μ-PtBA copolymer (Figure 10a), morphology of nanoparticles with an average diameter of approximately 45 nm were obtained, indicating the self-assembly of the PCL-μ-PtBA copolymer. In the PCL-μ-PSt and PCL-μ-PtBA copolymers, similar sphere shapes were both observed in the height and phase images which were ascribed to mainly observe the outer shell by using AFM. More detailed characterization of the miktoarm star polymers are currently underway. After deprotection, the height and phase images showed a flat and smooth surface due to the good miscibility of the PCL and PAA segments as shown in Figure 10b. We propose self-assembly microstructures of the miktoarm star block copolymers based on AFM studies. In the PCL-μ-PSt and PCL-μ-PtBA copolymers, each possesses incompatible segments and the PCL segment tends to undergo crystallization-driven aggregation. Figure 11 shows the schematically proposed representations of self-assembled microstructure from immiscible segments and homogeneous surface from miscible segments. As shown in Figure 11A, namely, the assembled nanostructures were comprising the semi-crystal PCL chains as the inner part and PSt or PtBA as the external part. High homogeneity of PCL and PAA blends through intermolecular hydrogen bonding has been reported [54]. In the PCL-μ-PAA, the results illustrated a homogenous surface of the miktoarm star copolymer (Figure 11B).

## 4. Conclusions

With the obtainment of quantitative chain-end-modification, high coupling efficiency (*x*_c_ ~ 0.94) of SA ATRC of PCL–Br with mono-modal MW distribution was successfully achieved with the aid of VBC at low temperature (VBC/PCL–Br/CuBr/Cu/PMDETA = 15/1/1/6/2.5 at 25 °C). From the MALDI-TOF MS, a coupling product with four VBC inserted units was revealed (i.e., PCL-VBC_4_-PCL) that can be rationally explained by the proposed SA ATRC mechanisms. By further proceeding with chain extensions of PCL-VBC_4_-PCL with St and tBA monomers via ATRP, controlled/living radical polymerization fashions were achieved and afforded unique PCL_2_-μ-PSt_4_ and PCL_2_-μ-PtBA_4_ six-arm star copolymers. Through DSC analysis, the *T*_g_ of amorphous segment and the *T*_m_ of PCL segment were detected for each μ-star copolymer in the first run, indicating the presence of semi-crystal PCL. However, the *T*_m_ of PCL was both disappeared in the second run that was rationally resulted from the nature of the star polymer with restricted chain mobility. Through TGA analysis, a multistep degrading profile was detected which was ascribed to the subsequent degradations of the different analogue of arms. By further conducting AFM measurements, the morphology of the pure PCL demonstrated obvious heterogeneity due to the amorphous and crystal macrophases. In PCL-μ-PSt and PCL-μ-PtBA copolymers, interesting nanoparticles were observed with an average diameter of approximately 250 and 45 nm, respectively. After deprotection of the *tert*-butyl group from the PCL-μ-PtBA copolymer, AFM images showed a flat and smooth surface which was due to the good miscibility of PCL and PAA segments. We herein showed facial and effective combinations of ROP, SA ATRC, and ATRP to synthesize PCL-based μ-star copolymers.

## Figures and Tables

**Table 1:** Summary of thermal properties of μ-star copolymers.

Sample	*T*_g_ (°C)	*T*_m_ (°C)	*x*_c_ (%) ^a^	*T*_d5%_ (°C) ^b^	Char Yield (wt %)
First run					
PCL-μ-PSt	81	60	5.5	150	2.8
PCL-μ-PtBA	40	55	2.6	151	1.2
Second run					
PCL-μ-PSt	80	n.d. ^c^	n.d.	–	–
PCL-μ-PtBA	36	n.d.	n.d.	–	–

^a^*x*_c_ (%) = percentage of crystallinity = Δ*H*_m_/Δ*H*_m_^o^ where Δ*H*_m_ is the experimental melting enthalpy of the samples and Δ*H*_m_^o^ is the standard melting enthalpy of the pure PCL (~136 J/g) [53]. ^b^ *T*_d5%_ = temperature of 5 wt % sample loss. ^c^ n.d. = none detected.

## Supplementary Materials

The following are available online at http://www.mdpi.com/2073-4360/10/8/858/s1, Table S1: Various inserted VBC units after SA ATRC of PCL–Br: estimated (Cal.) and measured (Exp.) *m*/*z* values in Figure 3, Table S2: Summary of rate constants based on initiators of EBiB, BzCl, and PEBr and monomers of MMA and St, Scheme S1: Reaction mechanisms of SA ATRC, Figure S1: FT-IR spectra of (a) PCL, (b) PCL-μ-PSt, and (c) PCL-μ-PtBA (co)polymers, Figure S2: FT-IR spectra of (a) PCL, (b) PCL-μ-PtBA, and (c) PCL-μ-PAA (co)polymers.
