# Supplementary material for: Synthesis of Poly(ε-caprolactone)-Based Miktoarm Star Copolymers through ROP, SA ATRC, and ATRP
Source: Polymers (Basel). 2018 Aug 2;10(8):858. doi: 10.3390/polym10080858 (PMC6403792; doi:10.3390/polym10080858)
Supplement: Supplementary file 1 [file polymers-10-00858-s001.pdf]

Synthesis of Poly(ε-caprolactone)-based Miktoarm Star Copolymers through ROP, SA ATRC, and ATRP

Venkatesan Sathesh, Jem-Kun Chen, Chi-Jung Chang, Zong-Cheng Chen, Yu-Chih Hsu, Yi-Shen Huang, and Chih-Feng Huang*

Supplementary Materials

Table of Contents

1. Supporting Tables **p. S2**

2. Supporting Scheme and Figures **p. S4**

3. References **p. S7**

Table S1. Various inserted VBC units after SA ATRC of PCL–Br: estimated (Cal.) and measured (Exp.) m/z values in Fig. 3 ($M_{n,GPC}$ = 5700, PDI = 1.18, x_c = 0.94).

Formula of (PCL_n)-VBC_m-$(PCL_n)/Na^+$ (based on n = 14)	Inserted VBC units (m)	m/z values	
		Cal.	Exp.
$C_{92}H_{154}O_{32}(C_9H_9Cl)_2/Na^+$	2	2099.61	
$C_{92}H_{154}O_{32}(C_9H_9Cl)_3/Na^+$	3	2252.23	
$C_{92}H_{154}O_{32}(C_9H_9Cl)_4/Na^+$	4	2404.85	2405.54
$C_{92}H_{154}O_{32}(C_9H_9Cl)_5/Na^+$	5	2557.47	

Table S2. Summary of rate constants based on initiators of EBiB, BzCl, and PEBr and monomers of MMA and St.

Rate constant	Value [M^{-1} s^{-1}]	Reference
$k_{a,EBiB}$	1.4	[1-3]
$k_{da,EBiB}$	1.9×10^{7}	[1-3]
$k_{t,MMA}$	6.1×10^{8}	[4]
$k_{a,BzCl}$	2.9×10^{-3}	[1-3]
$k_{da,BzCl}$	8.1×10^{5}	[1-3]
k_{i}	5.41×10^{3}	[5]
$k_{p,VBC}$	172.1	[1-3,6]
$k_{a,PEBr}$	8.8×10^{-2}	[1-3]
$k_{da,PEBr}$	2.7×10^{6}	[1-3]
$k_{t,VBC}$	3.99×10^{8}	[7]

k_a: ATRP activation rate constant; k_{da}: ATRP deactivation rate constant. The conditions to estimate the constants were described in the references (i.e. values of ethyl α-bromoisobutyrate (EBiB), benzyl chloride (BzCl) and 1-phenyl ethylbromide (PEBr) with CuBr/PMDETA measured in acetonitrile (MeCN) at 22 °C [1-3]; MMA: methyl methacrylate and St: styrene). k_i: rate constant for addition from methacrylic radical to St (value calculated from the frequency factor and activation energy) [5]. $k_{p,VBC}$: propagation rate constant of VBC; $k_{t,VBC}$: termination rate constant referred to the value of St.

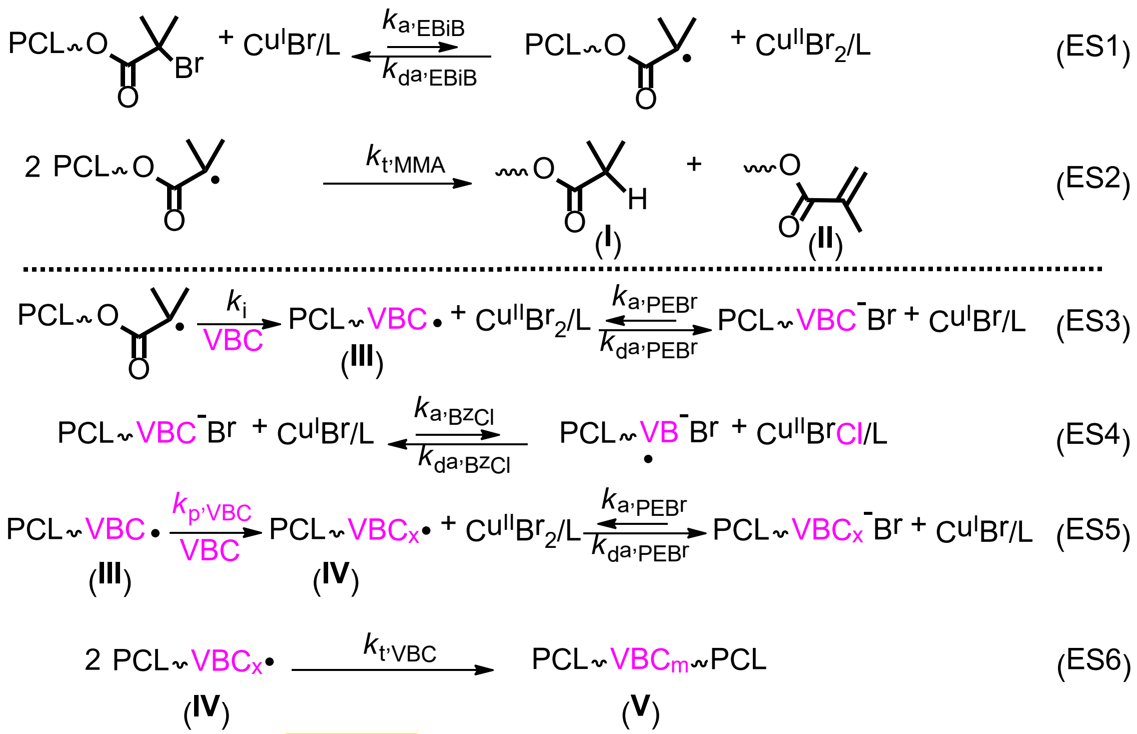

Scheme S1. Reaction mechanisms of SA ATRC.

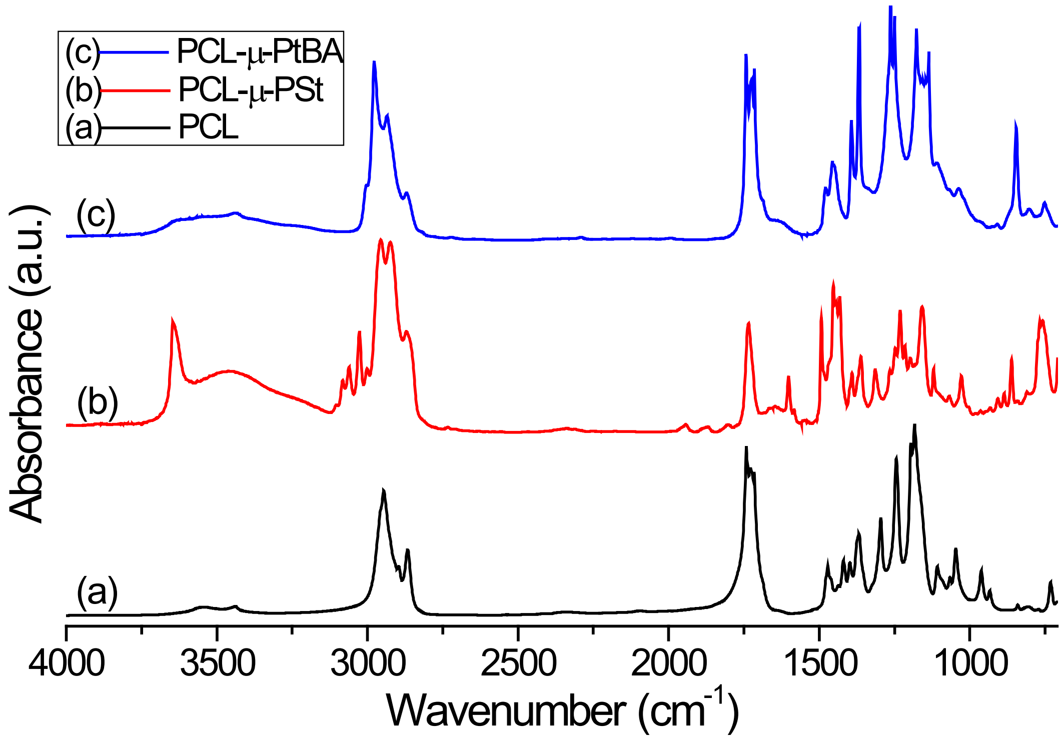

Figure S1. FT-IR spectra of (a) PCL, (b) PCL-μ-PSt, and (c) PCL-μ-PtBA (co)polymers.

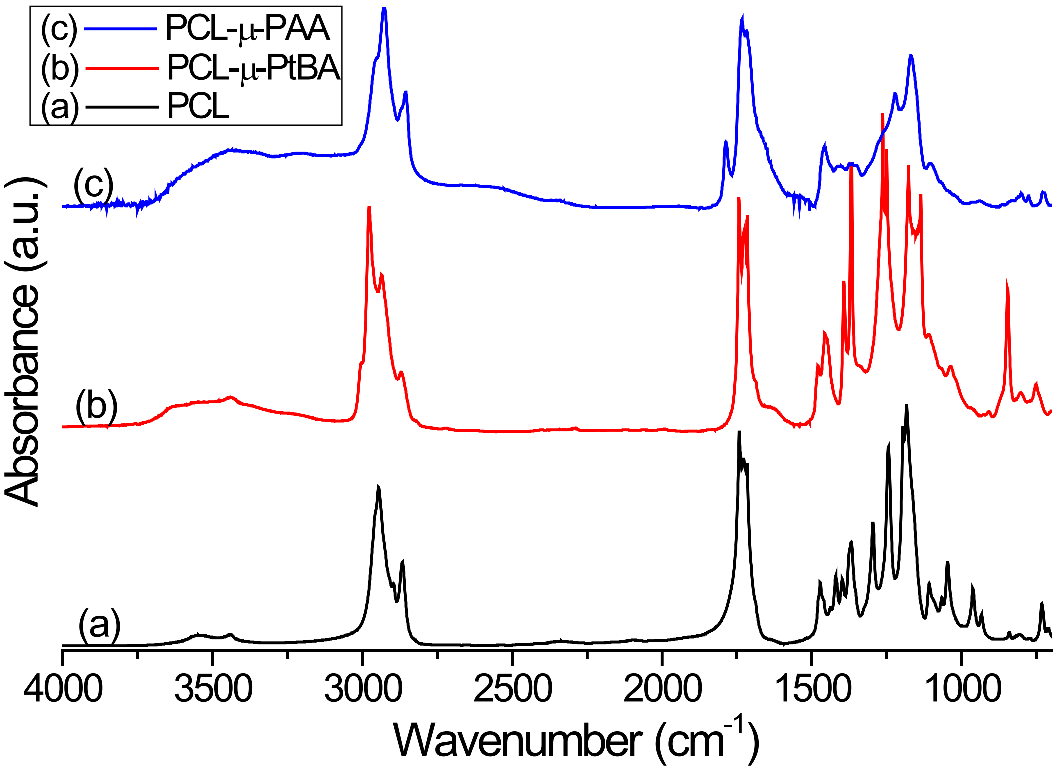

Figure S2. FT-IR spectra of (a) PCL, (b) PCL-μ-PtBA, and (c) PCL-μ-PAA (co)polymers.

3. References

1. Tang, W.; Kwak, Y.; Braunecker, W.; Tsarevsky, N.V.; Coote, M.L.; Matyjaszewski, K. Understanding atom transfer radical polymerization: Effect of ligand and initiator structures on the equilibrium constants. *J. Am. Chem. Soc.* **2008**, *130*, 10702-10713.
2. Tang, W.; Matyjaszewski, K. Kinetic modeling of normal ATRP, normal ATRP with $[Cu^{II}]_0$, reverse ATRP and SR&NI ATRP. *Macromol. Theor. Simul.* **2008**, *17*, 359-375.
3. Tang, W.; Tsarevsky, N.V.; Matyjaszewski, K. Determination of equilibrium constants for atom transfer radical polymerization. *J. Am. Chem. Soc.* **2006**, *128*, 1598-1604.
4. Barth, J.; Buback, M. SP-PLP-EPR investigations into the chain-length-dependent termination of methyl methacrylate bulk polymerization. *Macromol. Rapid Commun.* **2009**, *30*, 1805-1811.
5. Fischer, H.; Radom, L. Factors controlling the addition of carbon-centered radicals to alkenes-an experimental and theoretical perspective. *Angew. Chem. Int. Ed.* **2001**, *40*, 1340-1371.
6. Chen, Z.-C.; Chiu, C.-L.; Huang, C.-F. Tuning the solubility of copper complex in atom transfer radical self-condensing vinyl polymerizations to control polymer topology via one-pot to the synthesis of hyperbranched core star polymers. *Polymers* **2014**, *6*, 2552-2572.
7. Yoshikawa, C.; Goto, A.; Fukuda, T. Reactions of polystyrene radicals in a monomer-free atom transfer radical polymerization system. *e-Polymers* **2002**, no. 13.
